# Detection of quantitative trait loci affecting serum cholesterol, LDL, HDL, and triglyceride in pigs

**DOI:** 10.1186/1471-2156-12-62

**Published:** 2011-07-13

**Authors:** Muhammad Jasim Uddin, Do Ngoc Duy, Mehmet Ulas Cinar, Dawit Tesfaye, Ernst Tholen, Heinz Juengst, Christian Looft, Karl Schellander

**Affiliations:** 1 Institute of Animal Science, University of Bonn, Endenicher Allee 15, 53115 Bonn, Germany

**Keywords:** pig, QTL, serum lipids, F2 population

## Abstract

**Background:**

Serum lipids are associated with many serious cardiovascular diseases and obesity problems. Many quantitative trait loci (QTL) have been reported in the pig mostly for performance traits but very few for the serum lipid traits. In contrast, remarkable numbers of QTL are mapped for serum lipids in humans and mice. Therefore, the objective of this research was to investigate the chromosomal regions influencing the serum level of the total cholesterol (CT), triglyceride (TG), high density protein cholesterol (HDL) and low density protein cholesterol (LDL) in pigs. For this purpose, a total of 330 animals from a Duroc × Pietrain F2 resource population were phenotyped for serum lipids using ELISA and were genotyped by using 122 microsatellite markers covering all porcine autosomes for QTL study in QTL Express. Blood sampling was performed at approximately 175 days before slaughter of the pig.

**Results:**

Most of the traits were correlated with each other and were influenced by average daily gain, slaughter date and age. A total of 18 QTL including three QTL with imprinting effect were identified on 11 different porcine autosomes. Most of the QTL reached to 5% chromosome-wide (CW) level significance including a QTL at 5% experiment-wide (GW) and a QTL at 1% GW level significance. Of these QTL four were identified for both the CT and LDL and two QTL were identified for both the TG and LDL. Moreover, three chromosomal regions were detected for the HDL/LDL ratio in this study. One QTL for HDL on SSC2 and two QTL for TG on SSC11 and 17 were detected with imprinting effect. The highly significant QTL (1% GW) was detected for LDL at 82 cM on SSC1, whereas significant QTL (5% GW) was identified for HDL/LDL on SSC1 at 87 cM. Chromosomal regions with pleiotropic effects were detected for correlated traits on SSC1, 7 and 12. Most of the QTL identified for serum lipid traits correspond with the previously reported QTL for similar traits in other mammals. Two novel QTL on SSC16 for HDL and HDL/LDL ratio and an imprinted QTL on SSS17 for TG were detected in the pig for the first time.

**Conclusion:**

The newly identified QTL are potentially involved in lipid metabolism. The results of this work shed new light on the genetic background of serum lipid concentrations and these findings will be helpful to identify candidate genes in these QTL regions related to lipid metabolism and serum lipid concentrations in pigs.

## Background

Cholesterol (CT) is a lipid present in the cell membranes and transported in the bloodstream of all animals. CT is important for the synthesis of cell membrane and hormones and play vital role in the cell signaling process [1]. Cholesterol concentration in high density lipoprotein (HDL) and low density lipoprotein (LDL) are strong predictors for coronary heart disease and obesity. Higher concentration of LDL cholesterol and lower concentration of functional HDL are strongly associated with cardiovascular disease due to high risk of atherosclerosis [2]. LDL particles accumulated on the vessel walls to form lipid cores and cause inflammation that promotes atherosclerosis [3]. Triglyceride (TG) is the main constituent in vegetable oil and animal fat. The high level of triglyceride in the bloodstream is also linked to atherosclerotic heart disease [4] and pancreatitis [5]. The existence of a relevant amount of additive genetic variability for serum lipid traits in pigs has been demonstrated by Rothschild and Chapman [6] and Pond *et al*. [7], and subsequently corroborated in several divergent selection experiments [8,9].

The current release of the Pig QTLdb (http://www.animalgenome.org/cgi-bin/QTLdb/SS/viewmap) contains 5621 quantitative trait loci (QTL) representing 546 different traits (December, 2010) mostly for economically important traits like growth, carcass and meat quality, and reproduction. However, only two studies have so far been reported which have scanned the chromosomal regions influencing the serum lipid levels in pig. The first report of QTL for serum lipids [10] suggested a number of significant QTL in Duroc outbreed populations including two genome-wide significant QTL for HDL/LDL ratio (at 84 cM on SSC6) and for TG (at 23 cM on SSC4). Another study reported a total of 15 QTL including five novel QTL for serum lipids in a White Duroc × Erhualian intercross F2 population [11]. Hasler-Rapacz *et al*. [12] reported a locus on SSC2 linked to familial hypercholesterolemia in a Duroc group and showed the low-density-lipoprotein receptor gene (*LDLR*) as the candidate gene. In contrast, over the past two decades, lots of QTL have been identified in livestock, model organisms and human. Numerous QTL for serum lipids have been widely studied in human [13] and mice [14] such as QTL for TG which has been detected on every murine chromosome [15], and QTL for HDL on the mouse and human genomes are near ''saturated'' due to the concordance between the two species [16]. Moreover, beside as a source of food, the pig has tremendous biomedical importance as a model organism because of its closer proximity to human than most of the animal models including mice. The pig has the same size as humans as well as high anatomic and physiological similarities [17] and has the ability to be used in xenotransplantation [18]. The origin and distribution of the coronary arteries as well as the histological changes of growth and aging that apparently lead to atherosclerosis of aortas and coronary arteries in humans and pigs are also closely similar [19]. The common pathogenesis of atherosclerotic lesions in both humans and pigs [12] implies that pig could be the appropriate model for human atherosclerosis research. QTL study in different species, breeds and populations will deepen our understanding of the genetics behind lipid metabolism. The identified chromosomal regions will facilitate the identification of the potential candidate genes responsible for serum lipid concentrations in pigs which could be beneficial for human cardiovascular disease research. Therefore, the aim of this study was to identify the chromosomal regions affecting serum lipoproteins (TG, HDL, LDL, HDL/LDL and cholesterol) concentrations in a F2 Duroc × Pietrain resource population.

## Results

### Phenotypic variation of serum lipid traits

The descriptive statistics of the serum lipids traits measured in a Duroc × Pietrain (DUPI) F2 population are given in Table 1. The CT and HDL/LDL ratio was significantly correlated (*P *< 0.001) with all other traits. HDL also had significant correlation with TG levels. The HDL/LDL ratio was negatively correlated with CT (r = -0.42) and LDL (r = -0.69) concentration (Table 1). The GLM procedure in SAS allows dissection of the effects of genetic and environmental factors on these traits. Slaughter date, average daily gain (ADG) and age at slaughter significantly (*P *< 0.001) influenced the CT, HDL and LDL concentrations, whereas slaughter date and ADG had effect (*P *< 0.01) on TG concentrations. Only slaughter date had significant (*P *< 0.001) effect on the traits HDL/LDL ratio. This study could not detect any statistical significant effect of sex and litter size on any serum lipid traits.

**Table 1:** Descriptive statistics, correlation coefficient (above diagonal) and significance (below diagonal) of serum lipid traits

**Traits**^**a**^	N	**Mean ± SD**^**b**^	Minimum	Maximum	CT	HDL	TG	LDL	HDL/LDL
CT	330	81.18 ± 12.79	50.95	110.94		0.31	0.22	0.83	-0.42
HDL	330	23.68 ± 4.59	12.19	34.53	***		0.22	0.10	0.59
TG	330	40.18 ± 11.96	15.04	74.10	***	***		0.06	0.10
LDL	330	49.46 ± 11.26	25.53	78.12	***	NS	NS		-0.69
HDL/LDL	330	0.49 ± 0.14	0.21	1.28	***	***	***	***	

^a^Trait abbreviations: CT Cholesterol concentrations (mg/dL); HDL High Density Lipoprotein concentrations (mg/dL); TG Triglyceride concentrations (mg/dL); LDL Low Density Lipoprotein concentrations (mg/dL); HDL/LDL ratio between HDL and LDL concentrations, N = number of animals; NS not significant.

^b^Standard deviation; *** *P *< 0.001.

### QTL results for serum lipid traits

**Figure 1:**
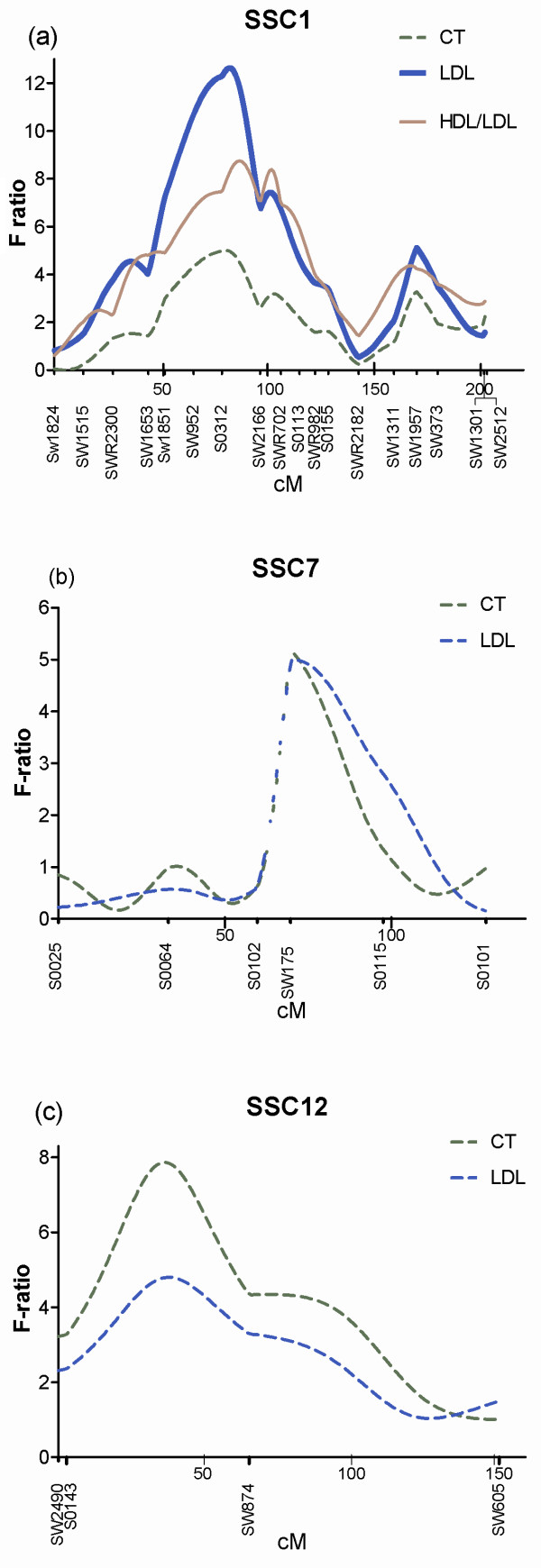
**F-ratio test statistics for the serum lipids traits on SSC1, SSC7 and SSC12**. The quantitative trait loci related to serum lipids traits with chromosome-wide significance at *P *< 0.05 (dotted curve; suggestive); experiment-wide level *P *< 0.05 (solid curve; significant) and *P *< 0.01 (solid, bold curve; highly significant) on SSC1 (Figure 1a), SSC7 (Figure 1b) and SSC12 (Figure 1c) estimated from data of the DUPI F2 resource population. Positions of the markers are indicated at the *x*-axis, F-values at the *y*-axis.

A total of 18 QTL were identified in 11 different autosomes, of which one QTL was highly significant (*P *< 0.01) at experiment-wide (GW) level, one QTL was significant (GW; *P *< 0.05), 15 QTL were suggestive (*P *< 0.05) at chromosome-wide (CW) level and only one QTL for TG on SSC2 was very close to the suggestive level with LOD 1.98 (Table 2). Most of the identified QTL showed higher additive effect. Interestingly, we identified a putative pleiotropic QTL region on SSC1 between the markers *S3012 *and *SW2166 *influencing the CT, LDL and HDL/LDL traits. The most significant QTL of this study was found for LDL concentrations (*F *value = 12.63) at 82 cM in this region on SSC1 explaining 8.12% of the phenotypic variations of the LDL concentrations. The GW significant QTL for HDL/LDL found at 87 cM on the same chromosomal region explained up to 5.78% of trait variances (Figure 1a). Two other possible pleiotropic QTL regions were detected at 70 cM on SSC7 (Figure 1b) and at 36-38 cM on SSC12 for CT and LDL concentration (Figure 1c). On SSC12, a suggestive chromosomal region was found (5%, CW) influencing the CT concentration close to the marker *S0143 *and contributed 5.21% of the trait variation. The suggestive (5% CW) QTL regions were identified at 0 cM on SSC3 for HDL, at 58 cM on SSC4 for HDL/LDL ratio and two regions on SSC6 at 66 cM and 99 cM for TG and CT, respectively.

**Table 2:** Summary of QTL for the serum lipid traits

**SSC**^**a**^	**Traits**^**b**^	**POS**^**c**^	***F*-value**^**d**^	LOD	**Var**^**e **^**(%)**	**a**^**f **^**± SE**	**d**^**g **^**± SE**	**Closest Markers**^**h**^
1	CT	80	5*	2.14	3.38	3.2 ± 1.01	0.22 ± 1.5	S0312 - SW2166
1	HDL/LDL	87	8.74**	3.69	5.78	-0.05 ± 0.01	0 ± 0.02	S0312 - SW2166
1	LDL	82	12.63***	5.26	8.12	4.62 ± 0.93	-0.94 ± 1.44	S0312 - SW2166
2	TG	205	4.64	1.98	3.14	-3.13 ± 1.03	-0.05 ± 1.62	SW1884 - SWR308
2	HDL^#^	118	4.44*	2.83	4.47	-0.03 ± 0.37	1.31 ± 0.59	SW240 - SW1564
3	HDL	0	4.54*	1.94	3.08	0.73 ± 0.37	-1.11 ± 0.52	SW72 - S0164
4	HDL/LDL	58	4.53*	1.94	3.08	-0.04 ± 0.01	-0.01 ± 0.02	S0001 - S0214
6	CT	99	5.73*	2.44	3.85	0.92 ± 1.01	-5.15 ± 1.63	S0059 - S0003
6	TG	66	4.5*	1.93	3.05	-2.91 ± 0.98	0.37 ± 1.53	S0087 - SW1067
7	CT	70	5.15*	2.2	3.48	2.74 ± 0.89	-1.41 ± 1.32	Sw175 - S0115
7	LDL	70	5*	2.13	3.38	2.52 ± 0.81	-0.67 ± 1.19	Sw175 - S0115
11	TG^#^	28	4.64*	2.96	4.66	-1.02 ± 0.98	0.61 ± 1.36	SW2008 - S0071
12	CT	36	7.86*	3.32	5.21	5.27 ± 1.63	-9.89 ± 4.03	S0143 - SW874
12	LDL	38	4.8*	2.05	3.25	3.86 ± 1.49	-6.6 ± 3.66	S0143 - SW874
13	LDL	97	6.17*	2.62	4.14	-0.17 ± 0.87	-4.76 ± 1.36	TNNC - SW398
16	HDL	81	4.51*	1.93	3.06	-1.28 ± 0.46	-1.25 ± 0.81	SW0026 - S0061
16	HDL/LDL	47	4.55*	1.95	3.09	-0.05 ± 0.02	-0.08 ± 0.04	S0111 - SW0026
17	TG^#^	72	4.44*	2.83	4.46	1.37 ± 1.52	9.67 ± 4.24	SW840 - SW2431

^a^Sus scrofa chromosome

^b^See Table 1

^c^Chromosomal position in Kosambi cM

^d^Significance of the QTL: * significant on a chromosome-wide level with *P *< 0.05; ** significant on a experiment-wide level with *P *< 0.05; *** significant on a experiment-wide level with *P *< 0.01

^e^The percentage of phenotypic variance explained by the QTL

^f^Additive effect and standard error. Positive values indicate the Duroc alleles result in higher values than Pietrain alleles; negative values indicate that Duroc alleles result in lower values than Pietrain alleles

^g^Dominance effect and standard error

^h^The closest markers were those markers around the peak, as near as possible (position of markers in cM)

^# ^The imprinting effect and standard error was detected for HDL (1.03 ± 0.38) on SSC2, for TG (-3.18 ± 0.9) on SSC11 and for TG (-4.6 ± 1.58) on SSC17. When both the additive and the imprinting effects are positive or negative, the paternal allele expresses (maternal imprinting); otherwise the maternal allele expresses (paternal imprinting)

The imprinting model revealed three imprinted QTL regions, a QTL for HDL on SSC2 (close to the marker *SW240*), and two QTL for TG on SSC11 (close to the marker *S0071*) and on SSC17 (close to the marker *SW2431*). QTL for HDL on SSC2 and for TG on SSC17 were paternally imprinted (maternally expressed) while the QTL for TG on SSC11 was maternally imprinted (paternally expressed) in this study (Table 2).

## Discussion

### Serum lipid concentrations

Despite being an excellent animal model for cholesterol related human diseases, the genetic architecture of serum lipids is not well understood in pigs [10]. The heritability of serum lipid traits are recorded from low to medium, for instance 0.22 for cholesterol levels [6], 0.18 to 0.30 for serum lipid concentrations [20]. The serum lipid concentrations can vary among breeds and age at sampling as shown by our results of serum lipid values which at 175 days in DUPI was slightly higher than the values recorded at 45 days but lower than the values recorded at 190 days of age in Duroc populations [10]. It has been reported in humans that serum lipid concentrations could vary with age [21]. Moreover, our results were close to the serum lipid concentrations reported in White Duroc and Erhualian crossbred population [11]. HDL, LDL and TG are all involved in lipid or cholesterol metabolism which supports our results since these traits were found to be highly positively correlated to each other (Table 1). We also found that HDL/LDL ratios are negatively correlated with CT and LDL which is in good agreement with previous report in pigs [10] for serum sampled at 190 days of age.

Serum lipid traits are known as quantitative traits which are influenced by both genetic and non genetic factors [6]. In the present study, slaughter date had influence on all the traits, and age at slaughter and average daily gain (ADG) were influencing the CT, LDL and HDL/LDL ratio in this study. Animals were fasted overnight before slaughter, which might affect the animal physiology and circulation system since Chen *et al*. [11] also reported that batches were affected the serum lipid traits. The impact of age at slaughter date on serum lipid concentrations is consistent with the previous report [10] that in pigs the serum lipid concentration is age-dependent. Sex is reported to have significant influence on the serum levels by several different studies in pigs [6,11,22] but in this study sex did not have any significant effect on serum lipids which is in accordance with Rothschild and Cahpman [6]. However, we found only slight difference in cholesterol concentrations between male and female animals in which males tended to have higher cholesterol concentrations compared to female (data not shown).

### QTL for serum lipid traits

Most of our identified QTL displayed high additive effect but still there were some over dominance QTL (Table 2). Duroc and Pietrain crosses are a good model for muscle and carcass trait studies [23,24] since Duroc and Pietrain are divergent for these traits [25]. However, to our knowledge, no such report is found with regards to serum lipid traits. It has been reported that the Duroc-sired pigs have more backfat and total fat tissues whereas the Pietrain-sired pigs have less backfat and total fat tissues [25]. Due to the unavailability of serum samples from pure breeds it was not possible for us to measure the serum lipid concentration in Duroc and Pietrain separately. The dominant QTL are difficult to explain since there are no reports about heterosis for serum lipid traits. Moreover, the low number of animals and family structure [23] of our F2 population should be considered. However, the existence of a relevant amount of additive genetic variability for serum lipid traits in pigs has been demonstrated by several studies [6-9] and some QTL for serum lipid traits with over dominance effect are also reported in pigs [11]. The genetic landscape of the QTL for serum lipid traits has been extensively investigated in humans, rats (http://rgd.mcw.edu/) and mice (http://www.informatics.jax.org/); but there are very few studies devoted to studying QTL for serum lipids in pigs. Most of our QTL are supported by homologous QTL regions recorded in other mammals (Table 3), especially in humans and mice. Detection of common QTL regions for similar traits among mammals could contribute to narrowing down the confidence regions that affect plasma lipid traits and subsequently to find positional candidate genes for cholesterol-related diseases such as heart disease, obesity and diabetes.

**Table 3:** Comparative positional analysis of identified QTL with serum lipid QTL in humans and mice and the putative candidate genes located on the QTL regions

SSC	Traits	POS (cM)	PCB at peak	HCHR	QTL in human	HCBP	Putative candidate genes	Mouse strain	QTL mouse peak
1	CT*	80	1q1.3 - 1.4	18q21 15q21-22	CT	18q21 [51]	*LIPC *[52]*, LIPG *[53]*, ACAA2 *[54]*, STARD6 *[55]	(B6 × C3H) F_2_ (B6 × DBA/2) F_2_ (B6 × CAST/2) F_2_ (B6 × C3H) F_2_	18-40cM 18-46cM 18-47cM 18-48cM
1	HDL/LDL**	87	1q1.5-1.6		HDL	15q21 [56], 15q22 [57]		(B6 × 129) F_2_	2-90 cM
1	LDL***	82	1q1.3 - 1.4		LDL-ApoB1	18q21.32 [58]			
2	TG	205	2q2.8-2.9	5q23-31	TG	5 q21.3 -q32 [59]	*STARD4 *[60]		
2	HDL*#	118	2p1.2- 2q1.2	11q13	HDL	11q12-13 [31]	*CILP, PBX4*	(MRL × SJL) F_2_ (B6 × C3H) F_2_	7-10 cM 7-19 cM
3	HDL*	0	3p1.5-p1.4	7q11	HDL TC	7q11.23 [61] 7q11.23 [62]	*FABP1*	(NZB × SM) F_2_ NZO × (SJL × NZO) (B6 × CAST) F_2_	5-61.cM 5-69cM 5-72 CM
4	HDL/LDL*	58	4q1.4-1.5	1q22-25	HDL	1q21-23 [63] 1q23-25 [64]; 1q24 [65], 1q25 [66]	*CYP7A1*[67,68]*;* *FABP5*[69]	(B6 × NZB11) F_2_	1-91 CM
6	CT*	99	6q2.3 - 2.4	1p36.3-36.1	LDL	1q36.31 [63] 1q36.13 [70]	*NR1H2* *WWOX *[71] LDLRAP1		
6	TG*	66	6q1.1 - 2.1	19q13	LDL, TG	19 q12 39 cM [72] 19q13.31 [51], [73]	*APOE *[74]; ANGPTL3		
7	CT, LDL*	70	7q1.4-71.5	15q24-28	LDL	15q26.1 [51]	*STARD5*		
11	TG*^#^	28	11p1.1 -1.2	13q12 -24	TG and HDL	13q13 [75]			
12	CT**	36	12p1.3-q1.2	17q21-25	CT/HDL	17q24 [76]	*WIPI1,*		
12	LDL*	38			LDL	17q24.2 [65]; 17q25.3 [77]	*THRA PEMT*		
13	LDL*	97	13q3.3 -3.5	3p31-21	LDL	3p25 [72]	*PCYT1A *[78]*; B4GALT4 *[79]	(C56BL/6JxDBA/2J)	3-27cM [80]
16	HDL*	81	16q1.4-2.1	5p14 -12	HDL	5p13.3 [81] 5p15.13 [82]		(PERAxDBA/2) F_2_ (PERAxI) F_2_	11-28 cM 11-28 cM
16	HDL/LDL*	47	16q1.3-1.4	5p14 -12	LDL	5p15.33 [64]			
17	TG*^#^	72	17q2.2-2.3	13q13	TG and HDL	13q13 [75]	*ACSS, PLTP*		

PCB, pig cytogenetic band; HCHR, human chromosome homologous regions; HCBP, human cytogenetic band.

Significance: Significance of the QTL: * significant on a chromosome-wide level with *P *< 0.05; ** significant on a experiment-wide level with *P *< 0.05; *** significant on a experiment-wide level with *P *< 0.01. Human homologous regions of the pig QTL were identified with the pig QTL database. ^# ^QTL with imprinting effect.

The most significant QTL found in the present study was the QTL for LDL at 82 cM on SSC1 (Table 2) which coincided with a QTL for ADG and two QTL for back fat thickness in pigs [26]. Moreover, in the same resource population, Liu *et al*. [23] found a 1% GW significance level QTL for ADG at 93 cM, supporting our findings that average daily weight gain is correlated to serum lipid levels. Gallardo *et al*. [10] also found a 1% CW significant QTL for LDL and a suggestive QTL for HDL/LDL ratio on SSC1. Additionally, we found a suggestive QTL for CT and a significant (5% GW) QTL for HDL/LDL ratio in this narrow region. These findings indicated that there are pleiotropic genes underlying this region affecting both fat and serum lipid concentrations in pigs. It is important to note that SSC1 was genotyped by 18 informative markers in this study which was advantageous to detect the peak of QTL with regards to markers (Figure 1a). This QTL region contained several potential candidate genes for serum lipid traits including endothelial lipase (*LIPG*), hepatic lipase (*LIPC*) and *ACAA2 *in humans and Stard6 in mice (Table 3). *LIPC *and *LIPG *are the members of lipase family genes and play significant roles in cholesterol metabolism especially in plasma HDL metabolism [27]. The *LIPC *gene encodes hepatic triglyceride lipase and has the capacity to catalyze the hydrolysis of phospholipids, mono-, di- and triglycerides [28]. Moreover, the promoter variant in *LIPC *is reported to be associated with elevated serum HDL cholesterol in humans and the variants of this gene are also associated with plasma HDL [29]. Animals that over express *LIPG *are reported to show decreased HDL cholesterol levels and those lacking *LIPG *show elevated levels of HDL cholesterol [30]. Furthermore, this region is homologous to human chromosome HSA18q21 and HSA15q21 harbouring QTL for CT and QTL for HDL in humans (Table 3). The region is also homologous with a 40 - 48 cM region on mouse chromosome 18 (MUS18) where a number of QTL for HDL are reported at 40, 46, 47 and 48 cM in different strains [31].

We found a QTL region for TG at 205 cM on SSC2q2.8-2.9 which was very close to the suggestive level and the confidence interval of this QTL overlaps with the previously reported QTL for TG in pigs [11]. This region is homologous with the QTL region for TG on HSA5q21.3-32 (Table 3). Moreover, a paternally imprinted QTL for HDL is identified at 118 cM very approximate to the marker *SW240 *on SSC2 and very close to this region Liu *et al*. [23] reported a imprinting QTL for backfat thickness in the same population. A remarkable number of imprinting QTL have been identified on SSC2 (http://igc.otago.ac.nz/) such as imprinting QTL for backfat thickness [32] and average backfat [33] in pigs. *IGF2 *which is reported to be have strong imprinting effects on muscle mass and fat deposition [34], is located on SSC2 within the confidence interval of our identified imprinting QTL region for HDL on SSC2. Kadlecova *et al*. [35] suggested that polymorphism in the *IGF2 *gene might have effects on lipid metabolism in rats. Moreover, the *IGF2 *gene is reported to play a central role in atherosclerosis in a mouse model [36]. A suggestive QTL for HDL is identified at 0 cM on SSC3 and close to this region, Gallardo *et al*. [10] reported QTL for cholesterol at 27 cM and 15 cM, and for HDL at 28 cM in pigs. This region corresponds to HSA7q11 that showed a consistent association with atherosclerosis disease in human [37]. It has been well established that HDL-cholesterol is the key member of the reverse transport process of cholesterol flowing from peripheral tissue to the liver in mammals and is greatly negatively associated with atherosclerotic disease [11]. A suggestive chromosomal region for HDL/LDL ratio was detected at the pericentromeric region on SSC4 in this study (Table 2). HDL/LDL cholesterol ratio is reported to be an excellent predictor to asses the risk of heart disease and to monitor the effectiveness of lipid lowering therapies [38]. The peak QTL region harbors fatty acid binding protein 5 (*FABP5*) on SSC4 which is known to have functions in lipid metabolism; the knockdown of *FABP5 *decreased the cellular cholesterol (Table 3). The cholesterol 7 alpha-hydroxylase (*CYP7A1*) gene is also located in our region of interest on SSC4 and is reported to be responsible for coding a rate-limiting enzyme in the synthesis of bile acid from cholesterol. Moreover, several studies revealed the association of this gene with the plasma LDL levels in humans (Table 3).

Two neighbour QTL regions on SSC6 were identified for TG and CT within 33 cM very close to the marker *SW1067 *and *S0059*, respectively. Apolipoprotein E (*ApoE*) is an important gene for the HDL metabolism and is located within the confidence interval of these QTL on SSC6. This gene contributes more to normal cholesterol variability than any other gene identified in cholesterol metabolism [39]. A QTL in the corresponding region on HSA19q13 influenced both the TG levels and adiposity in human (Table 3). Close to the marker *SW175*, a possible pleotropic QTL for both the LDL and CT is found on SSC7 at 70 cM in this study (Figure 1b). LDL and CT were positively (r = 0.83, *P *< 0.001) correlated in the studied population. Pleiotropic QTL for HDL and CT close to the marker *SW632 *are also identified by Gallardo *et al*. [10] in Duroc populations. This QTL region is homologous to HAS15q26 affecting the LDL in humans (Table 3). A suggestive QTL for CT was identified at 38 cM on SSC12 and contributes 5.2% of phenotypic variation of total cholesterol (Table 2). Two cM apart from this QTL, another suggestive QTL was found for LDL (Figure 1c), which can be assumed as a possible pleiotropic QTL in this study. For these two QTL, alleles that increase phenotypic variation were inherited from the Duroc breed. Gallardo *et al*. [10] also found a QTL for TG and HDL/LDL ratio very close to our identified region on SSC12. Corresponding to this region, HSA17q24 is reported to influence the plasma CT and HDL in humans (Table 3).

This study identified a suggestive QTL for LDL at 97 cM on SSC13 (Table 2). Based on the identified QTL for HDL, CT, LDL and HDL/LDL ratio with pleotropic effects between 72-79 cM in Duroc pigs, Gallardo *et al*. [10] speculated to have several interesting loci such as phosphate cytidylyltransferase 1, choline, α-isoform (*PCYT1A*) and the UDP-Gal:betaGlcNAc beta 1,4- galactosyltransferase, polypeptide4 (*B4GALT4*) in this region (Table 3). In this study, two novel QTL were identified on SSC16 for HDL at 81 cM and HDL/LDL ratio at 47 cM, which are homologous to HSA5q31 and HSA5p14-12, respectively. QTL for HDL is reported on HSA5p13.3 and HSA5p15.13 in humans (Table 3). To the best of our knowledge, the identified pig genomic regions influencing the serum lipid concentration are described for the first time. A novel imprinting QTL for TG at 72 cM is detected on SSC17 in this study (Table 2) and several imprinted QTL have been reported in this chromosomal region such as QTL for longissimus fat content, marbling [40] and for average daily gain [33] in pigs.

## Conclusions

The results of this work enrich genetic landscapes of serum lipid traits in pigs. Since cholesterol, lipoproteins and triglyceride are considered as strong predictors for atherosclerosis disease, the identification of the QTL regions influencing these traits is an important step for understanding the genetics behind and to scan for the candidate genes related to serum lipid-related diseases. However, before addressing the candidate genes in the identified QTL regions, it is important to note that the confidence intervals of the QTL were large and could contain many genes affecting the serum lipid traits. The SSC1q1.3-q1.6 region harbouring significant QTL for serum lipid traits requires fine mapping to detect potential candidate genes in this region. Two novel QTL regions on SSC16 for lipoprotein traits have been identified and are worthwhile of further research.

## Methods

### Resource population

The animal population used for the evaluation of serum lipoprotein traits included three generations (P, F1 and F2) of Duroc × Pietrain family as described earlier [23,24,41,42]. A total of 330 F2 pigs were used in this study. All animals were kept at the Frankenforst experimental research farm at the University of Bonn (Germany) according to the rules of German performance stations (Zentralverband der Deutschen Schweineproduktion (ZDS): Richtlinie für die Stationsprüfung auf Mastleistung, Schlachtkörperwert und Fleischbeschaffenheit beim Schwein, 10.12.2003). The F2 pigs were fed the same diet *ad libitum *during the whole test period and were slaughtered at approximately 105 kg live weight at around the age of 25 weeks. Samples from the tail were collected within the first week after birth for DNA isolation. Blood samples were taken at approximately 175 days of age before slaughter with fasting but free access to overnight. Serum was isolated from blood and stored at -80°C until use.

### Measurement of lipoproteins levels

All serum lipid traits were assayed by ELISA according to the manufacturer's procedure (BioAssay Systems, USA) and read at 570 nm by ELISA Plate Reader (DYNEX Technologies. Inc, USA). In brief, total cholesterol and TG concentrations were measured using Enzy Chrom™ cholesterol assay kit (cat. E2CH-100) and Enzy Chrom™ triglyceride assay kit (cat. ETGA-200), respectively. The slopes used for the calculation of CT and TG levels were determined by linear regression fitted using serial diluted concentrations. 10 μL of serum samples and 50 μL of working reagent were added to 96 well plates. The plates were tapped well and incubated for 30 minutes at room temperature before reading the optical density (OD). The CT and TG concentrations were calculated based on the slopes of the standard line, OD value of samples and blank according to the manufacturer's formula. For HDL measurement, colorimetric procedure using EnzyChrom™ HDL and LDL/VLDL Assay Kit (cat. EHDL-100) was used. Briefly, equal volume (20 μL) of serum and precipitation reagent were mixed by vortexing. After centrifugation, 24 μL of the supernatant was pipetted carefully into a clean tube and 96 μL assay buffer was added. Then, equal amount (50 μL) of the mixture, assay buffer and cholesterol standard were transferred separately into 96-well plates. Finally, 50 μL of working reagent was added to each well. The plates were then tapped well and read at 570 nm in ELISA plate reader. HDL concentrations were calculated from OD of sample, blank and standards following manufacturer's formula. LDL concentrations were calculated following Freidewald's formula (mg/dL) [43].

### Statistical analysis

The data were analysed using the SAS software package version 9.2 (SAS Institute Inc., Cary, NC, USA) for a detailed description of the data structure. Descriptive statistics and correlations among these traits were calculated by the PROC MEANS and PROC CORR procedures (Table 1). Generalised linear models (PROC GLM) were used to identify any possible effect of sire, dam, sex, average daily weight gain, ages at slaughter, slaughter date and litter size on the serum lipid concentrations. The phenotypic data followed approximately a normal distribution and were used for linkage analysis.

### Marker analysis

A linkage map with the total length of 2159.3 cM and an average marker interval of 17.7 cM was constructed [42]. P, F1 and F2 animals of the Duroc × Pietrain (DUPI) population were genotyped previously [23], [44] at 122 markers loci covering all porcine autosomes. Marker positions and details of genotyping procedures were given in Liu *et al*. [23] and for SSC1 in Grosse-Brinkhaus *et al*. [44]. These markers were mainly selected from the USDA/MARC map (http://www.marc.usda.gov) and most are available in Sscrofa5 (http://www.ncbinlmnihgov/projects/mapview/map_searchcgi?taxid=9823) and Sscrofa9 (http://www.ensemblorg/Sus_scrofa/Info/Index). Genotyping, electrophoresis, and allele determination were done using a LI-COR 4200 Automated Sequencer including the software OneDScan (Scanalytics). The CE8000 sequencer (Beckman Coulter, USA) was used for genotyping of SSC1 and SSC18. Allele and inheritance genotyping errors were checked using Pedcheck software (version 1.1) [45]. The relative positions of the markers were assigned using the build, twopoint and fixed options of CRIMAP software (version 2.4) [46]. Recombination units were converted to map distances using the Kosambi mapping function. Marker information content and segregation distortion were tested by following Knott *et al*. [47].

### QTL analysis

F2 QTL interval mapping was performed using the web-based program QTL Express [48] based on a least square regression method. Single-QTL analyses were carried out and imprinting model was also applied. The basic QTL regression model used in the present study was:

where: y_i _= phenotype of the i^th ^offspring; μ = overall mean; F_i _= Fixed effect (slaughter date, i from 1 to 40); β = regression coefficient on the covariate; cov_i _= covariate (average daily weight gain and age at slaughter (in days) were covariates for CT, HDL and LDL; average daily weight gain was covariates for TG; and HDL/LDL ratio had no covariate); c_ai _= additive coefficient of the i^th ^individual at a putative QTL in the genome; c_di _= dominant coefficient of the i^th ^individual at a putative QTL in the genome; a = additive effects of a putative QTL; d = dominant effects of a putative QTL; and ε_i _= residual error.

The presence of imprinting effects was tested by adding a third (imprinting) effect (i) into the model [47] using QTL Express [48]. Chromosome- (CW) and experiment-wide (GW) significance thresholds were determined using 1000 permutations [49]. The empirical 95% confidence intervals (CI) and flanking markers for the QTL positions were obtained with 1000 re-sampling steps [50]. The phenotype variation that was explained by a QTL was calculated by the following equation.

Where, MS_R _is the mean of square of the reduced model, MS_F _is the mean of square of the full model.

To identify positional candidate genes underlying QTL, syntenic regions corresponding to QTL were defined by integrated comparative RH map at http://animalgenome.org/cgi-bin/QTLdb/SS/viewmap, and candidate genes were determined via the UCSC Genome Browser (http://genome.ucsc.edu/cgi-bin/hgGateway).

## Competing interests

The authors declare that there is no competing financial or other interest in relation to this work.

## Authors' contributions

MJU and DND performed the experiments, analysed data and prepared and edited the manuscript. MUC arranged the experiment facilities and edited the manuscript. HJ was responsible for sampling. DT, ET and CL edited the manuscript with MJU and DND. KS criticized the experimental design, supervised this study and edited the manuscript. All authors read and approved the final manuscript.
